# Supplementary material for: Perspectives and Attitudes of Newer New Jersey High School Teachers towards Cleaning, Sanitizing, and Disinfecting Consumer Products Used in School Classrooms
Source: Int J Environ Res Public Health. 2024 Feb 10;21(2):211. doi: 10.3390/ijerph21020211 (PMC10887922; doi:10.3390/ijerph21020211)
Supplement: Supplementary file 1 [file ijerph-21-00211-s001.zip › ijerph-2808330-supplementary.pdf]

(v.1 Aug2021) Rutgers NJ Safe Schools Program New Work-Based Learning Supervising Teachers Cohort - 1st Survey (Parts I and II, and Demographics)

Hazard Concern

*1) Overall, how safe do you feel in your school workplace?

- ◯ Very safe [Value=1]
- ◯ Moderately safe [Value=2]
- ◯ Not really safe [Value=3]
- ◯ Unsure/I do not know [Value=4]
- ◯ I prefer not to answer this question [Value=5]

*2) Overall, how confident are you in your students knowing what to do if an emergency occurs at school?

- ◯ Very confident [Value=1]
- ◯ Moderately confident [Value=2]
- ◯ Not really confident [Value=3]
- ◯ Unsure/I do not know [Value=4]
- ◯ I prefer not to answer this question [Value=5]

———————————Page Break———————————

Built or Physical School Environment

*3) In your opinion, which aspect of the school built environment most exacerbates the potential for a school emergency on your school's property? Please choose your top response.

- ◯ Unmonitored entrances [Value=1]
- ◯ Lack of security systems (for example, no video cameras or no security officers) [Value=2]
- ◯ Hidden stairwells [Value=3]
- ◯ Dense foliage around school [Value=4]
- ◯ I prefer not to answer this question [Value=5]

---

*4) What do you believe can be done by the school to better help school personnel and students prepare for potential emergency situations? Please choose your top response.

- ◯ Provide assistance for developing emergency plans [Value=1]
- ◯ Provide information/training on preparedness [Value=2]
- ◯ Organize and conduct drills [Value=3]
- ◯ Provide equipment and supplies [Value=4]
- ◯ I prefer not to answer this question [Value=5]

---

5) Please rank the initiatives you find the most effective in preparing teachers and students to prepare for potential emergency situations.
1= most effective 4= least effective

- ☐ Provide assistance to the school for developing emergency plans
- ☐ Provide relevant information and trainings for teachers and students on preparedness
- ☐ Organize and conduct drills during the school day
- ☐ Provide equipment and supplies

———————————Page Break———————————

Safety and Health Protocols and Training

*6) To the best of your knowledge and observation, are employees who wear respirators at your workplace(s) trained and medically cleared to do so?

- ◯ Yes, employees are trained and medically cleared to use respirators. [Value=1]

◯ No, employees are not trained and medically cleared to use respirators. [Value=2]
◯ N/A or not applicable, we only use face coverings and masks. [Value=3]
◯ I prefer not to answer this question [Value=4]

*7) Does your employer have a training protocol to clearly delineate the differences between cloth face coverings, surgical masks, filtering face pieces and respirators?

◯ Yes [Value=1]
◯ No [Value=2]
◯ N/A [Value=3]
◯ I prefer not to answer this question [Value=4]

*8) Based on your experience, with your career cluster(s) and program pathway(s), do you believe most employers have effective safety and health programs?

◯ Yes, most employers have effective safety and health programs. [Value=1]
◯ No, most employers do not have effective safety and health programs. [Value=2]
◯ I prefer not to answer this question [Value=3]

————————————Page Break————————————

Attitudes and Attributes

*9) How concerned are you about your health and safety at your school campus?

◯ Very Concerned [Value=1]
◯ Concerned [Value=2]
◯ Neutral [Value=3]
◯ Unconcerned [Value=4]
◯ Very Unconcerned [Value=5]
◯ I prefer not the answer this question [Value=6]

*10) How concerned are you about your health and safety in your assigned school classroom/office space?

◯ Very Concerned [Value=1]
◯ Concerned [Value=2]
◯ Neutral [Value=3]
◯ Unconcerned [Value=4]
◯ Very Unconcerned [Value=5]
◯ I prefer not the answer this question [Value=6]

The following question is asking about cleaning (removes dirt and organic matter from surfaces), sanitizing (kills bacteria on surfaces), disinfection (kills bacteria and viruses on surfaces) products.

For further information please see: https://www.epa.gov/coronavirus/whats-difference-between-products-disinfect-sanitize-and-clean-surfaces

	a.) cleaning products	b.) sanitizing products	c.) disinfection products
*11) Have you ever required medical care because of using _____ at school?	--Select--<br>- Yes [Value=1]<br>- No [Value=2]<br>- N/A [Value=3]<br>- I prefer not to answer this question [Value=4]	--Select--<br>- Yes [Value=1]<br>- No [Value=2]<br>- N/A [Value=3]<br>- I prefer not to answer this question [Value=4]	--Select--<br>- Yes [Value=1]<br>- No [Value=2]<br>- N/A [Value=3]<br>- I prefer not to answer this question [Value=4]

————————————Page Break————————————

Perceptions on Safety and Health (S&H)

	a.) in the school classrooms	b.) in eating areas (cafeteria, student and faculty lounges, etc.)	c.) inside the gym	d.) outside at fields (sit on stands/bleachers, use of sports equipment).
*12) I have the right to be healthy and feel safe:	--Select--<br>- Strongly Agree [Value=1]<br>- Agree [Value=2]<br>- Neutral [Value=3]<br>- Disagree [Value=4]<br>- Strongly	--Select--<br>- Strongly Agree [Value=1]<br>- Agree [Value=2]<br>- Neutral [Value=3]<br>- Disagree [Value=4]<br>- Strongly	--Select--<br>- Strongly Agree [Value=1]<br>- Agree [Value=2]<br>- Neutral [Value=3]<br>- Disagree [Value=4]<br>- Strongly	--Select--<br>- Strongly Agree [Value=1]<br>- Agree [Value=2]<br>- Neutral [Value=3]<br>- Disagree [Value=4]<br>- Strongly

	Disagree [Value=5] - I prefer not to answer this question [Value=6]	Disagree [Value=5] - I prefer not to answer this question [Value=6]	Disagree [Value=5] - I prefer not to answer this question [Value=6]	Disagree [Value=5] - I prefer not to answer this question [Value=6]

*13) In school, whose S&H should be top priority?

- ◯ Students [Value=1]
- ◯ Teachers [Value=2]
- ◯ Other staff [Value=3]
- ◯ Principals [Value=4]
- ◯ All of the above [Value=5]
- ◯ I prefer not to answer this question [Value=6]

14) At school, who is responsible for S&H?

- ☐ O&M staff [Checked=1]
- ☐ Principal [Checked=1]
- ☐ Teacher [Checked=1]
- ☐ Other staff [Checked=1]

*15) You can use either antibacterial or plain regular hand soap to properly wash your hands

- ◯ True [Value=1]
- ◯ False [Value=2]
- ◯ I prefer to not answer this question [Value=3]

*16) How long is it recommended to wash your hands with soap and water (in seconds)?

- ◯ 5-10 [Value=1]
- ◯ 10-15 [Value=2]
- ◯ 20-25 [Value=3]
- ◯ 30 [Value=4]
- ◯ I prefer not to answer the question [Value=5]

*17) You can use hand sanitizer as a replacement to washing your hands with soap and water.

- ◯ True [Value=1]
- ◯ False [Value=2]
- ◯ I prefer to not answer this question [Value=3]

*18) If you wear gloves when using chemical products, do you also need to wash your hands afterwards?

- ◯ Yes [Value=1]
- ◯ No [Value=2]
- ◯ I prefer to not answer this question [Value=3]

19) What percent of the thousands of chemicals in cleaning/disinfection products are classified as toxic by the U.S.? (Write in between 0-99%)

[ ]

*20) Low- and odor-free products are often healthier than scented products for school indoor air quality.

- ◯ True [Value=1]
- ◯ False [Value=2]
- ◯ I prefer to not answer this question [Value=3]

*21) Custodians and facilities operations/maintenance staff are more likely to leave their job due to occupational related allergies and health problems than office workers.

- ◯ True [Value=1]
- ◯ False [Value=2]
- ◯ I prefer to not answer this question [Value=3]

*22) How concerned are you about health effects from ingredients in cleaning products?

- ◯ Very Concerned [Value=1]

- Concerned [Value=2]
- Neutral [Value=3]
- Unconcerned [Value=4]
- Very Unconcerned [Value=5]
- I prefer not the answer this question [Value=6]

*23) How concerned are you about health effects from ingredients in disinfection products?

- Very Concerned [Value=1]
- Concerned [Value=2]
- Neutral [Value=3]
- Unconcerned [Value=4]
- Very Unconcerned [Value=5]
- I prefer not the answer this question [Value=6]

————————————————Page Break————————————————

Products Purchased for Your Use at School

The following three questions are asking about cleaning (removes dirt and organic matter from surfaces), sanitizing (kills bacteria on surfaces), disinfection (kills bacteria and viruses on surfaces) products.

For further information please see: https://www.epa.gov/coronavirus/whats-difference-between-products-disinfect-sanitize-and-clean-surfaces

24) Where do you buy most of your cleaning products?

- Grocery Stores (for example, alphabetically: Kings, ShopRite, Stop and Shop, Wegmans, etc.) [Checked=1]
- Pharmacies (for example, alphabetically: CVS, RiteAid, Walgreens, etc.) [Checked=1]
- Big Box Stores (for example, alphabetically: Costco, Sam's Club, Target, Walmart, etc.) [Checked=1]
- By mail/Online (for example, alphabetically: Amazon) [Checked=1]
- Hardware Stores (for example, alphabetically: Ace Hardware, Home Depot, Lowes, etc.) [Checked=1]
- I do not know, the school provides supplies [Checked=1]

25) Where do you buy most of your disinfection products?

- Grocery Stores (for example, alphabetically: Kings, ShopRite, Stop and Shop, Wegmans, etc.) [Checked=1]
- Pharmacies (for example, alphabetically: CVS, RiteAid, Walgreens, etc.) [Checked=1]
- Big Box Stores (for example, alphabetically: Costco, Sam's Club, Target, Walmart, etc.) [Checked=1]
- By mail/Online (for example, alphabetically: Amazon) [Checked=1]
- Hardware Stores (for example, alphabetically: Ace Hardware, Home Depot, Lowes, etc.) [Checked=1]
- I do not know, the school provides supplies [Checked=1]

26) Where do you buy most of your sanitation products?

- Grocery Stores (for example, alphabetically: Kings, ShopRite, Stop and Shop, Wegmans, etc.) [Checked=1]
- Pharmacies (for example, alphabetically: CVS, RiteAid, Walgreens, etc.) [Checked=1]
- Big Box Stores (for example, alphabetically: Costco, Sam's Club, Target, Walmart, etc.) [Checked=1]
- By mail/Online (for example, alphabetically: Amazon) [Checked=1]
- Hardware Stores (for example, alphabetically: Ace Hardware, Home Depot, Lowes, etc.) [Checked=1]
- I do not know, the school provides supplies [Checked=1]

	a.) cleaning products	b.) sanitizing products	c.) disinfection products
*27) When you buy a _____ to use, how often do you read ingredients on the label?	--Select-- - Very Often [Value=1]<br>- Often [Value=2]<br>- Sometimes [Value=3]<br>- Not Often [Value=4]<br>- Never [Value=5]<br>- N/A [Value=6]<br>- I prefer not to answer this question [Value=7]	--Select-- - Very Often [Value=1]<br>- Often [Value=2]<br>- Sometimes [Value=3]<br>- Not Often [Value=4]<br>- Never [Value=5]<br>- N/A [Value=6]<br>- I prefer not to answer this question [Value=7]	--Select-- - Very Often [Value=1]<br>- Often [Value=2]<br>- Sometimes [Value=3]<br>- Not Often [Value=4]<br>- Never [Value=5]<br>- N/A [Value=6]<br>- I prefer not to answer this question [Value=7]
*28) When buying a ____ to use, how often do you look for labels indicating the product is made with natural, non-toxic or eco-friendly ingredients?	--Select--	--Select--	--Select--

		a.) cleaning products	b.) sanitizing products	c.) disinfection products
29)	Before purchasing the _____, do you look it up on a healthy product app or website?	--Select-- - Yes [Value=1] - No [Value=2] - NA [Value=3] - I prefer not to answer this question [Value=4]	--Select-- - Yes [Value=1] - No [Value=2] - NA [Value=3] - I prefer not to answer this question [Value=4]	--Select-- - Yes [Value=1] - No [Value=2] - NA [Value=3] - I prefer not to answer this question [Value=4]

Page Break

Demographic Information

***30)** School County:
Please select the NJ county of your school district/school.
Note: If you work for State of NJ agency (e.g., NJDCF, NJDOE), select "statewide" or last option below.

- Atlantic [Value=1]
- Bergen [Value=2]
- Burlington [Value=3]
- Camden [Value=4]
- Cape May [Value=5]
- Cumberland [Value=6]
- Essex [Value=7]
- Gloucester [Value=8]
- Hudson [Value=9]
- Hunterdon [Value=10]
- Mercer [Value=11]
- Middlesex [Value=12]
- Monmouth [Value=13]
- Morris [Value=14]
- Ocean [Value=15]
- Passaic [Value=16]
- Salem [Value=17]
- Somerset [Value=18]
- Sussex [Value=19]
- Union [Value=20]
- Warren [Value=21]
- statewide [Value=22]

31) Race and Ethnicity: Please choose the best option from the list below (listed in alphabetical order), or "other" (write in).

- American Indian or Alaskan Native [Checked=1]
- Native Hawaiian or Other Asian-Pacific Islander [Checked=1]
- Middle Eastern/North African [Checked=1]
- Hispanic Asian [Checked=1]
- Hispanic Black [Checked=1]
- Hispanic White [Checked=1]
- Non-Hispanic Asian [Checked=1]
- Non-Hispanic Black [Checked=1]
- Non-Hispanic White [Checked=1]
- I prefer not to answer this question [Checked=1]
- Other (please specify) [Checked=1]

***32)** Gender Identity

- Male [Value=1]
- Female [Value=2]
- Transgender Man [Value=3]
- Transgender Women [Value=4]
- Non-Binary [Value=5]
- I prefer not to answer this question [Value=6]
- Other (please specify) [Value=7]

*33) Birth Year

--Select--

- 2000 [Value=1]
- 1999 [Value=2]
- 1998 [Value=3]
- 1997 [Value=4]
- 1996 [Value=5]
- 1995 [Value=6]
- 1994 [Value=7]
- 1993 [Value=8]
- 1992 [Value=9]
- 1991 [Value=10]
- 1990 [Value=11]
- 1989 [Value=12]
- 1988 [Value=13]
- 1987 [Value=14]
- 1986 [Value=15]
- 1985 [Value=16]
- 1984 [Value=17]
- 1983 [Value=18]
- 1982 [Value=19]
- 1981 [Value=20]
- 1980 [Value=21]
- 1979 [Value=22]
- 1978 [Value=23]
- 1977 [Value=24]
- 1976 [Value=25]
- 1975 [Value=26]
- 1974 [Value=27]
- 1973 [Value=28]
- 1972 [Value=29]
- 1971 [Value=30]
- 1970 [Value=31]
- 1969 [Value=32]
- 1968 [Value=33]
- 1967 [Value=34]
- 1966 [Value=35]
- 1965 [Value=36]
- 1964 [Value=37]
- 1963 [Value=38]
- 1962 [Value=39]
- 1961 [Value=40]
- 1960 [Value=41]
- 1959 [Value=42]
- 1958 [Value=43]
- 1957 [Value=44]
- 1956 [Value=45]
- 1955 [Value=46]
- 1954 [Value=47]
- 1953 [Value=48]
- 1952 [Value=49]
- 1951 [Value=50]
- 1950 [Value=51]
- Other (please specify) [Value=52]

Other:

*34) Number of Years Teaching in NJ

--Select--

- 1 [Value=1]
- 2 [Value=2]
- 3 [Value=3]
- 4 [Value=4]
- 5 [Value=5]
- 6 [Value=6]
- 7 [Value=7]
- 8 [Value=8]
- 9 [Value=9]
- 10 [Value=10]
- 11 [Value=11]
- 12 [Value=12]
- 13 [Value=13]
- 14 [Value=14]
- 15 [Value=15]
- 16 [Value=16]
- 17 [Value=17]
- 18 [Value=18]
- 19 [Value=19]
- 20 [Value=20]
- 21 [Value=21]
- 22 [Value=22]
- 23 [Value=23]
- 24 [Value=24]

- 25 [Value=25]
- 26 [Value=26]
- 27 [Value=27]
- 28 [Value=28]
- 29 [Value=29]
- 30 [Value=30]
- 31 [Value=31]
- 32 [Value=32]
- 33 [Value=33]
- 34 [Value=34]
- 35 [Value=35]
- Other (please specify) [Value=36]

Other:

*35) Number of Years of Teaching Overall

--Select--

- 1 [Value=1]
- 2 [Value=2]
- 3 [Value=3]
- 4 [Value=4]
- 5 [Value=5]
- 6 [Value=6]
- 7 [Value=7]
- 8 [Value=8]
- 9 [Value=9]
- 10 [Value=10]
- 11 [Value=11]
- 12 [Value=12]
- 13 [Value=13]
- 14 [Value=14]
- 15 [Value=15]
- 16 [Value=16]
- 17 [Value=17]
- 18 [Value=18]
- 19 [Value=19]
- 20 [Value=20]
- 21 [Value=21]
- 22 [Value=22]
- 23 [Value=23]
- 24 [Value=24]
- 25 [Value=25]
- 26 [Value=26]
- 27 [Value=27]
- 28 [Value=28]
- 29 [Value=29]
- 30 [Value=30]
- 31 [Value=31]
- 32 [Value=32]
- 33 [Value=33]
- 34 [Value=34]
- 35 [Value=35]
- Other (please specify) [Value=36]

Other:

*36) What is the highest education degree completed (choose from options presented)?

○ Bachelor's degree (BA, BS or AB) [Value=1]
○ Master's degree (e.g., MA, MS, M.Ed) [Value=2]
○ Doctoral degree (e.g., DrPH, Ed.D, PhD, PsychD, ScD) [Value=3]
○ Other (please specify) [Value=4]

Other:

*37) How many years of post secondary education (after high school) have you completed?

--Select--

- 1 [Value=1]
- 2 [Value=2]
- 3 [Value=3]
- 4 [Value=4]
- 5 [Value=5]
- 6 [Value=6]
- 7 [Value=7]
- 8 [Value=8]
- 9 [Value=9]
- 10 [Value=10]
- 11 [Value=11]
- 12 [Value=12]
- 13+ [Value=13]
- Other (please specify) [Value=14]

Other:

Skip Logic
Skip to survey **[#193755]**, question **[#1]**

——————————————Skip Logic——————————————

——————————————Automatic Page Break——————————————

(v.1 Aug2021) Rutgers NJ Safe Schools Program New Work-Based Learning Supervising Teachers Cohort - 1st Survey (Parts I and II, and Demographics)

Thank you!

For maximum confidentiality, please close this window.

Copyright © 2001-2022 PsychData®, LLC. All rights reserved.

(v.1 Aug2021) Rutgers NJ Safe Schools Program New Work-Based Learning Supervising Teachers Cohort - 2nd Survey [Follow-up on Safety, Health and Wellness (physical and mental)]

Place of Work and Time Worked

*1) On average, how many days per week do you work at this school?

--Select--

- 1 [Value=1]
- 2 [Value=2]
- 3 [Value=3]
- 4 [Value=4]
- 5 [Value=5]
- 6 [Value=6]
- 7 [Value=7]
- I prefer not to answer [Value=20]
- Other (please specify) [Value=19]

Other:

*2) On average, how many hours do you work per day in your assigned classroom/office at this school each week?

--Select--

- 1 [Value=1]
- 2 [Value=2]
- 3 [Value=3]
- 4 [Value=4]
- 5 [Value=5]
- 6 [Value=6]
- 7 [Value=7]
- 8 [Value=8]
- 9 [Value=9]
- 10 [Value=10]
- 11 [Value=11]
- 12 [Value=12]
- I prefer not to answer [Value=20]
- Other (please specify) [Value=19]

Other:

Page Break

Safety & Health (of Employee)

*3) How important of a concern is your safety and health while working at your school?

◯ Very Important [Value=1]
◯ Important [Value=2]
◯ Neutral [Value=3]
◯ Unimportant [Value=4]
◯ Very Unimportant [Value=5]
◯ I prefer not to answer [Value=6]
◯ N/A [Value=7]

*4) How important of a concern is your safety and health while working in your assigned classroom/office?

◯ Very Important [Value=1]
◯ Important [Value=2]
◯ Neutral [Value=3]
◯ Unimportant [Value=4]
◯ Very Unimportant [Value=5]
◯ I prefer not to answer [Value=6]
◯ N/A [Value=7]

Page Break

Symptoms (of Employee)

*5) Have you ever had any of the following respiratory health problems after working in this school: runny nose, itchy/watery eyes, trouble breathing or headache?

◯ Yes [Value=1]
◯ No [Value=2]
◯ I'm not sure [Value=3]
◯ I prefer not to answer [Value=4]

Question Logic
If **[Yes]** is selected, then skip to question **[#6]**
If **[No]** is selected, then skip to question **[#7]**
If **[I'm not sure]** is selected, then skip to question **[#7]**
If **[I prefer not to answer]** is selected, then skip to question **[#7]**

Page Break

*6) Did you need to seek medical attention in response to the severity of any symptoms? (runny nose, itchy/watery eyes, trouble breathing and/or headache)

◯ Yes [Value=1]
◯ No [Value=2]
◯ I prefer not to answer [Value=3]

*7) If this is your 1st year at this school, did you experience any of these symptoms at your prior school or job? (runny nose, itchy/watery eyes, trouble breathing and/or headache)

◯ Yes [Value=1]
◯ No [Value=2]
◯ I'm not sure [Value=3]
◯ N/A [Value=5]
◯ I prefer not to answer [Value=4]

*8) Do any of these symptoms disappear if you are away from work more than a day? (runny nose, itchy/watery eyes, trouble breathing and/or headache)

◯ Yes [Value=1]
◯ No [Value=2]
◯ I'm not sure [Value=3]
◯ N/A [Value=5]
◯ I prefer not to answer [Value=4]

Page Break

Ventilation

*9) Is there a classroom or shop ventilation system?

◯ Yes [Value=1]
◯ No [Value=2]
◯ I'm not sure [Value=5]
◯ N/A [Value=3]
◯ I prefer not to answer [Value=4]

*10) Can the windows be opened in the school room you spend most time in?

◯ Yes [Value=1]
◯ No [Value=2]
◯ I'm not sure [Value=5]
◯ N/A [Value=3]
◯ I prefer not to answer [Value=4]

*11) Is there a ceiling fan in the salon?

◯ Yes [Value=1]
◯ No [Value=2]
◯ I'm not sure [Value=5]
◯ N/A [Value=3]
◯ I prefer not to answer [Value=4]

Page Break

Personal Protective Equipment (Products Use)

*12) When performing cleaning/disinfection services, do you wear gloves?

◯ Yes [Value=1]
◯ No [Value=2]
◯ N/A [Value=3]

◯ I prefer not to answer [Value=4]

***13)** When performing cleaning/disinfection services, do you wear a mask?

◯ Yes [Value=1]
◯ No [Value=2]
◯ N/A [Value=3]
◯ I prefer not to answer [Value=4]

——————————Page Break——————————

Training

***14)** Was training offered on how to handle cleaning/sanitizing/disinfecting products at your workplace?

◯ Yes [Value=1]
◯ No [Value=2]
◯ I'm not sure [Value=3]
◯ N/A [Value=5]
◯ I prefer not to answer [Value=4]

Question Logic
If **[Yes]** is selected, then skip to question **[#15]**
If **[No]** is selected, then skip to question **[#16]**
If **[I'm not sure]** is selected, then skip to question **[#16]**
If **[N/A]** is selected, then skip to question **[No logic applied]**
If **[I prefer not to answer]** is selected, then skip to question **[#16]**

——————————Page Break——————————

***15)** Was the training in your primary language?

◯ Yes [Value=1]
◯ No [Value=2]
◯ I'm not sure [Value=3]
◯ N/A [Value=5]
◯ I prefer not to answer [Value=4]

***16)** Are you aware of U.S. government resources, from USDOL-OSHA?

◯ Yes [Value=1]
◯ No [Value=2]
◯ I prefer not to answer [Value=3]

***17)** Are you aware of Hazard Communication Standard and/or Safety Data Sheets?

◯ Yes [Value=1]
◯ No [Value=2]
◯ I prefer not to answer [Value=3]

***18)** Are you aware of NJ Safe Schools' Cosmetology/Right to Know website pages?

◯ Yes [Value=1]
◯ No [Value=2]
◯ I prefer not to answer [Value=3]

——————————Page Break——————————

Personal Nutrition

***19)** How often do you now eat processed snacks/foods?

◯ Rarely [Value=1]
◯ Daily [Value=2]
◯ Weekly [Value=3]
◯ Monthly [Value=4]

◯ N/A [Value=5]

◯ I prefer not to answer [Value=6]

How often do you eat from (take-out) and/or at restaurants, for:

	Daily	Three or more times a week	One to two times a week	N/A	I prefer not to answer
*20) Breakfast?	◯ [Value=1]	◯ [Value=2]	◯ [Value=3]	◯ [Value=4]	◯ [Value=5]
*21) Lunch?	◯ [Value=1]	◯ [Value=2]	◯ [Value=3]	◯ [Value=4]	◯ [Value=5]
*22) Dinner?	◯ [Value=1]	◯ [Value=2]	◯ [Value=3]	◯ [Value=4]	◯ [Value=5]

Page Break

Personal Sleep Hygiene

*23) How many hours do you sleep per night on average on regular school days?

--Select--

- Less than 5 hours [Value=1]
- 5 - 6 hours [Value=2]
- 7 - 8 hours [Value=3]
- 9 hours or more [Value=4]
- I prefer not to answer [Value=5]

*24) How many hours do you sleep per night on average on weekends and holidays/vacation days?

--Select--

- Less than 5 hours [Value=1]
- 5 - 6 hours [Value=2]
- 7 - 8 hours [Value=3]
- 9 hours or more [Value=4]
- I prefer not to answer [Value=5]

Please answer yes, no or I prefer to not answer to the following:

	Yes	No	I'm not sure	I prefer not to answer
*25) I wake up in middle of night.	◯ [Value=1]	◯ [Value=2]	◯ [Value=4]	◯ [Value=3]
*26) I find it difficult to wake up in the morning on a work/school day.	◯ [Value=1]	◯ [Value=2]	◯ [Value=4]	◯ [Value=3]

Page Break

Personal Physical Health

*27) I regularly use PPE or personal protective equipment (in the previous school year 2020-2021).

◯ Yes [Value=1] ◯ No [Value=2] ◯ I'm not sure [Value=3] ◯ I prefer not to answer [Value=4]

*28) I regularly use PPE or personal protective equipment (in the current school year 2021-2022).

◯ Yes [Value=1] ◯ No [Value=2] ◯ I'm not sure [Value=4] ◯ I prefer not to answer [Value=3]

Question Logic
If **[Yes]** is selected, then skip to question **[after #28, Matrix Text]** (See "Edit Logic" for details)
If **[No]** is selected, then skip to question **[#32]**
If **[I'm not sure]** is selected, then skip to question **[#32]**
If **[I prefer not to answer]** is selected, then skip to question **[#32]**

Page Break

Do you use the following forms of PPE to protect yourself?

	Yes	No	I'm not sure	I prefer not to answer
*29) Mask/face coverings?	◯ [Value=1]	◯ [Value=2]	◯ [Value=3]	◯ [Value=4]
*30) Gloves?	◯ [Value=1]	◯ [Value=2]	◯ [Value=3]	◯ [Value=4]
*31) Protective eyewear?	◯ [Value=1]	◯ [Value=2]	◯ [Value=3]	◯ [Value=4]

*32) Did you maintain consistent aerobic exercise habits during the course of the COVID-19 pandemic?

◯ Yes [Value=1] ◯ No [Value=2] ◯ I'm not sure [Value=3] ◯ I prefer not to answer [Value=4]

*33) Have you maintained similar aerobic exercise habits during the pandemic when compared to before the pandemic?

◯ Yes [Value=1] ◯ No [Value=2] ◯ I'm not sure [Value=3] ◯ I prefer not to answer [Value=4]

Question Logic
If **[Yes]** is selected, then skip to question **[#34]**
If **[No]** is selected, then skip to question **[after #34, Text]** (See "Edit Logic" for details)
If **[I'm not sure]** is selected, then skip to question **[after #34, Text]** (See "Edit Logic" for details)
If **[I prefer not to answer]** is selected, then skip to question **[after #34, Text]** (See "Edit Logic" for details)

Page Break

*34) Was there an increase or decrease in your aerobic exercise habits during the pandemic when compared to before the pandemic?

◯ Increase [Value=1] ◯ Decrease [Value=2] ◯ I'm not sure [Value=3] ◯ I prefer not to answer [Value=4]

Page Break

Personal Mental Health

In your opinion, who is responsible for supporting the mental well-being of each of the following:

	other school educational professionals, including administrators and staff	school guidance/academic counselors	school health and behavioral health staff, including school psychologists	community health and behavioral health providers	N/A	I prefer not to answer
*35) School educational professionals	◯ [Value=1]	◯ [Value=2]	◯ [Value=3]	◯ [Value=4]	◯ [Value=5]	◯ [Value=6]
*36) O&M staff and other types of staff	◯ [Value=1]	◯ [Value=2]	◯ [Value=3]	◯ [Value=4]	◯ [Value=5]	◯ [Value=6]
*37) Students	◯ [Value=1]	◯ [Value=2]	◯ [Value=3]	◯ [Value=4]	◯ [Value=5]	◯ [Value=6]

Do you know someone who had a positive test result for SARS-CoV-2 (COVID-19)?

	Yes	No	I'm not sure	I prefer not to answer
*38) Yourself?	◯ [Value=1]	◯ [Value=2]	◯ [Value=3]	◯ [Value=4]
*39) An immediate family member?	◯ [Value=1]	◯ [Value=2]	◯ [Value=3]	◯ [Value=4]
*40) An extended family member?	◯ [Value=1]	◯ [Value=2]	◯ [Value=3]	◯ [Value=4]
*41) A friend or work colleague?	◯ [Value=1]	◯ [Value=2]	◯ [Value=3]	◯ [Value=4]

Do you know someone who has died from COVID-19?

	Yes	No	I'm not sure	I prefer not to answer
An immediate family member?	◯	◯	◯	◯

***42)**	[Value=1]	[Value=2]	[Value=3]	[Value=4]
***43)** An extended family member?	◯ [Value=1]	◯ [Value=2]	◯ [Value=3]	◯ [Value=4]
***44)** A friend or work colleague?	◯ [Value=1]	◯ [Value=2]	◯ [Value=3]	◯ [Value=4]

***45)** Would you recommend the available mental health services to a colleague?

◯ Yes [Value=1]
◯ No [Value=2]
◯ I'm not sure [Value=3]
◯ N/A [Value=5]
◯ I prefer not to answer [Value=4]

***46)** If you have used the mental health services in the school before, on a scale of 1-10 with 10 being the most satisfied, how satisfied were you with the services?

--Select--

- 1 [Value=1]
- 2 [Value=2]
- 3 [Value=3]
- 4 [Value=4]
- 5 [Value=5]
- 6 [Value=6]
- 7 [Value=7]
- 8 [Value=8]
- 9 [Value=9]
- 10 [Value=10]
- I prefer not to answer [Value=11]

47) As a teacher, please select the mental health supports you would like to see at your school as schools transition back to full-time in-person learning? Please select/choose all that apply to you at this time.

☐ Improve mental health literacy with psychological first aid or training in how to talk about mental health. [Checked=1]
☐ Increase availability of mental health and counseling services. [Checked=1]
☐ Expanding virtual counseling services and tele-health services. [Checked=1]
☐ Involvement in discussions about what might change and what they need. [Checked=1]
☐ Consistent communication to reduce uncertainty and build emotional support. [Checked=1]
☐ I prefer not to answer [Checked=1]
☐ Other (please specify) [Checked=1]

48) Self-care means taking the time to do things that help you live well and improve both your physical health and mental health. What self-care practices have you implemented in your day-to-day life since the beginning of the COVID-19 pandemic?

☐ Getting regular exercise. [Checked=1]
☐ Eating healthy. [Checked=1]
☐ Staying hydrated. [Checked=1]
☐ Making sleep a priority. Stick to a schedule, and make sure you're getting enough sleep. [Checked=1]
☐ Trying/practicing a relaxing activity. [Checked=1]
☐ Practicing gratitude and positivity. [Checked=1]
☐ Staying connected with friends or family members. [Checked=1]
☐ I prefer not to answer [Checked=1]
☐ Other (please specify) [Checked=1]

Page Break

Demographics

***49)** County
Please select the NJ county of your school district/school.
Note: If you work for State of NJ agency (e.g., NJDCF, NJDOE), select "statewide" or last option below.

◯ Atlantic [Value=3]
◯ Bergen [Value=4]
◯ Burlington [Value=5]
◯ Camden [Value=6]

◯ Cape May [Value=7]
◯ Cumberland [Value=8]
◯ Essex [Value=9]
◯ Gloucester [Value=10]
◯ Hudson [Value=11]
◯ Hunterdon [Value=12]
◯ Mercer [Value=13]
◯ Middlesex [Value=14]
◯ Monmouth [Value=15]
◯ Morris [Value=16]
◯ Ocean [Value=17]
◯ Passaic [Value=18]
◯ Salem [Value=19]
◯ Somerset [Value=20]
◯ Sussex [Value=21]
◯ Union [Value=22]
◯ Warren [Value=23]
◯ Statewide [Value=2]

50) Race and Ethnicity
Please choose the best option from the list below (listed in alphabetical order), or "other" (write in).

☐ American Indian or Alaskan Native [Checked=1]
☐ Middle Eastern/North African [Checked=1]
☐ Native Hawaiian or Other Asian-Pacific Islander [Checked=1]
☐ Hispanic Asian [Checked=1]
☐ Hispanic Black [Checked=1]
☐ Hispanic White [Checked=1]
☐ Non-Hispanic Asian [Checked=1]
☐ Non-Hispanic Black [Checked=1]
☐ Non-Hispanic White [Checked=1]
☐ I prefer not to answer [Checked=1]
☐ Other (please specify) [Checked=1]

***51)** Gender

◯ Male [Value=1]
◯ Female [Value=2]
◯ Transgender Woman [Value=5]
◯ Transgender Man [Value=6]
◯ Non-binary [Value=8]
◯ I prefer not to answer [Value=3]
◯ Other [Value=4]

Skip Logic
Skip to survey **[#193757]**, question **[#1]**

————————————Skip Logic————————————

————————————Automatic Page Break————————————

(v.1 Aug2021) Rutgers NJ Safe Schools Program New Work-Based Learning Supervising Teachers Cohort - 2nd Survey [Follow-up on Safety, Health and Wellness (physical and mental)]

Thank you!

For maximum confidentiality, please close this window.

Copyright © 2001-2022 PsychData®, LLC. All rights reserved.

Late 2022/2022-2023 School Year Follow-up (v.1 Aug2021) "Rutgers NJ Safe Schools Program New Work-Based Learning Supervising Teachers Cohort - Survey

Safety and Health Protocols and Training

*1) Overall, how safe do you feel in your school workplace?

- ◯ Very safe [Value=1]
- ◯ Moderately safe [Value=2]
- ◯ Not really safe [Value=3]
- ◯ Unsure/I do not know [Value=4]
- ◯ I prefer not to answer this question [Value=5]

————————————————Page Break————————————————

*2) How concerned are you about your health and safety at your school campus?

- ◯ Very Concerned [Value=1]
- ◯ Concerned [Value=2]
- ◯ Neutral [Value=3]
- ◯ Unconcerned [Value=4]
- ◯ Very Unconcerned [Value=5]
- ◯ I prefer not the answer this question [Value=6]

*3) How concerned are you about your health and safety in your assigned school classroom/office space?

- ◯ Very Concerned [Value=1]
- ◯ Concerned [Value=2]
- ◯ Neutral [Value=3]
- ◯ Unconcerned [Value=4]
- ◯ Very Unconcerned [Value=5]
- ◯ I prefer not the answer this question [Value=6]

*4) Based on your experience, with your career cluster(s) and program pathway(s), do you believe most employers have effective safety and health programs?

- ◯ Yes, most employers have effective safety and health programs. [Value=1]
- ◯ No, most employers do not have effective safety and health programs. [Value=2]
- ◯ I prefer not to answer this question [Value=3]

————————————————Page Break————————————————

*5) To the best of your knowledge and observation, are employees who wear respirators at your workplace(s) trained and medically cleared to do so?

- ◯ Yes, employees are trained and medically cleared to use respirators. [Value=1]
- ◯ No, employees are not trained and medically cleared to use respirators. [Value=2]
- ◯ N/A or not applicable, we only use face coverings and masks. [Value=3]
- ◯ I prefer not to answer this question [Value=4]

*6) Does your employer have a training protocol to clearly delineate the differences between cloth face coverings, surgical masks, filtering face pieces and respirators?

- ◯ Yes [Value=1]
- ◯ No [Value=2]
- ◯ N/A [Value=3]
- ◯ I prefer not to answer this question [Value=4]

The following question is asking about cleaning (removes dirt and organic matter from surfaces), sanitizing (kills bacteria on surfaces), disinfection (kills bacteria and viruses on surfaces) products.

For further information please see: https://www.epa.gov/coronavirus/whats-difference-between-products-disinfect-sanitize-and-clean-surfaces

	a.) cleaning products	b.) sanitizing products	c.) disinfection products
*7) Have you ever required medical care because of using _____ at school?	--Select-- - Yes [Value=1]<br>- No [Value=2]<br>- N/A [Value=3]<br>- I prefer not to answer this question [Value=4]	--Select-- - Yes [Value=1]<br>- No [Value=2]<br>- N/A [Value=3]<br>- I prefer not to answer this question [Value=4]	--Select-- - Yes [Value=1]<br>- No [Value=2]<br>- N/A [Value=3]<br>- I prefer not to answer this question [Value=4]

——————————————————Page Break——————————————————

Perceptions on Safety and Health (S&H)

	a.) in the school classrooms	b.) in eating areas (cafeteria, student and faculty lounges, etc.)	c.) inside the gym	d.) outside at fields (sit on stands/bleachers, use of sports equipment).
*8) I have the right to be healthy and feel safe:	--Select-- - Strongly Agree [Value=1]<br>- Agree [Value=2]<br>- Neutral [Value=3]<br>- Disagree [Value=4]<br>- Strongly Disagree [Value=5]<br>- I prefer not to answer [Value=6]	--Select-- - Strongly Agree [Value=1]<br>- Agree [Value=2]<br>- Neutral [Value=3]<br>- Disagree [Value=4]<br>- Strongly Disagree [Value=5]<br>- I prefer not to answer [Value=6]	--Select-- - Strongly Agree [Value=1]<br>- Agree [Value=2]<br>- Neutral [Value=3]<br>- Disagree [Value=4]<br>- Strongly Disagree [Value=5]<br>- I prefer not to answer [Value=6]	--Select-- - Strongly Agree [Value=1]<br>- Agree [Value=2]<br>- Neutral [Value=3]<br>- Disagree [Value=4]<br>- Strongly Disagree [Value=5]<br>- I prefer not to answer [Value=6]

*9)
In school, whose S&H should be top priority?
- ◯ Students [Value=1]
- ◯ Teachers [Value=2]
- ◯ Other staff [Value=3]
- ◯ Principals [Value=4]
- ◯ All of the above [Value=5]
- ◯ I prefer not to answer this question [Value=6]

10) At school, who is responsible for S&H?
- ☐ O&M staff [Checked=1]
- ☐ Principal [Checked=1]
- ☐ Teacher [Checked=1]
- ☐ Other staff [Checked=1]

*11) You can use either antibacterial or plain regular hand soap to properly wash your hands
- ◯ True [Value=1]
- ◯ False [Value=2]
- ◯ I prefer to not answer this question [Value=3]

*12) How long is it recommended to wash your hands with soap and water (in seconds)?
- ◯ 5-10 [Value=1]
- ◯ 10-15 [Value=2]
- ◯ 20-25 [Value=3]
- ◯ 30 [Value=4]
- ◯ I prefer not to answer the question [Value=5]

*13) You can use hand sanitizer as a replacement to washing your hands with soap and water.
- ◯ True [Value=1]
- ◯ False [Value=2]
- ◯ I prefer to not answer this question [Value=3]

*14) If you wear gloves when using chemical products, do you also need to wash your hands afterwards?
- ◯ Yes [Value=1]
- ◯ No [Value=2]

◯ I prefer to not answer this question [Value=3]

————————————————Page Break————————————————

*15) What percent of the thousands of chemicals in cleaning/disinfection products are classified as toxic by the U.S.? (Write in between 0-99%)

[ ]

*16) Low- and odor-free products are often healthier than scented products for school indoor air quality.

◯ True [Value=1]
◯ False [Value=2]
◯ I prefer to not answer this question [Value=3]

*17) Custodians and facilities operations/maintenance staff are more likely to leave their job due to occupational related allergies and health problems than office workers.

◯ True [Value=1]
◯ False [Value=2]
◯ I prefer to not answer this question [Value=3]

*18) How concerned are you about health effects from ingredients in cleaning products?

◯ Very Concerned [Value=1]
◯ Concerned [Value=2]
◯ Neutral [Value=3]
◯ Unconcerned [Value=4]
◯ Very Unconcerned [Value=5]
◯ I prefer not the answer this question [Value=6]

*19) How concerned are you about health effects from ingredients in disinfection products?

◯ Very Concerned [Value=1]
◯ Concerned [Value=2]
◯ Neutral [Value=3]
◯ Unconcerned [Value=4]
◯ Very Unconcerned [Value=5]
◯ I prefer not the answer this question [Value=6]

————————————————Page Break————————————————

Products Purchased for Your Use at School

The following three questions are asking about cleaning (removes dirt and organic matter from surfaces), sanitizing (kills bacteria on surfaces), disinfection (kills bacteria and viruses on surfaces) products.

For further information please see: https://www.epa.gov/coronavirus/whats-difference-between-products-disinfect-sanitize-and-clean-surfaces

20) Where do you buy most of your cleaning products?

☐ Grocery Stores (for example, alphabetically: Kings, ShopRite, Stop and Shop, Wegmans, etc.) [Checked=1]
☐ Pharmacies (for example, alphabetically: CVS, RiteAid, Walgreens, etc.) [Checked=1]
☐ Big Box Stores (for example, alphabetically: Costco, Sam's Club, Target, Walmart, etc.) [Checked=1]
☐ By mail/Online (for example, alphabetically: Amazon) [Checked=1]
☐ Hardware Stores (for example, alphabetically: Ace Hardware, Home Depot, Lowes, etc.) [Checked=1]
☐ I do not know, the school provides supplies [Checked=1]

21) Where do you buy most of your disinfection products?

☐ Grocery Stores (for example, alphabetically: Kings, ShopRite, Stop and Shop, Wegmans, etc.) [Checked=1]
☐ Pharmacies (for example, alphabetically: CVS, RiteAid, Walgreens, etc.) [Checked=1]
☐ Big Box Stores (for example, alphabetically: Costco, Sam's Club, Target, Walmart, etc.) [Checked=1]
☐ By mail/Online (for example, alphabetically: Amazon) [Checked=1]
☐ Hardware Stores (for example, alphabetically: Ace Hardware, Home Depot, Lowes, etc.) [Checked=1]
☐ I do not know, the school provides supplies [Checked=1]

22) Where do you buy most of your sanitation products?

- ☐ Grocery Stores (for example, alphabetically: Kings, ShopRite, Stop and Shop, Wegmans, etc.) [Checked=1]
- ☐ Pharmacies (for example, alphabetically: CVS, RiteAid, Walgreens, etc.) [Checked=1]
- ☐ Big Box Stores (for example, alphabetically: Costco, Sam's Club, Target, Walmart, etc.) [Checked=1]
- ☐ By mail/Online (for example, alphabetically: Amazon) [Checked=1]
- ☐ Hardware Stores (for example, alphabetically: Ace Hardware, Home Depot, Lowes, etc.) [Checked=1]
- ☐ I do not know, the school provides supplies [Checked=1]

	a.) cleaning products	b.) sanitizing products	c.) disinfection products
***23)** When you buy a _____ to use, how often do you read ingredients on the label?	--Select-- - Very Often [Value=1]<br>- Often [Value=2]<br>- Sometimes [Value=3]<br>- Not Often [Value=4]<br>- Never [Value=5]<br>- N/A [Value=6]<br>- I prefer not to answer this question [Value=7]	--Select-- - Very Often [Value=1]<br>- Often [Value=2]<br>- Sometimes [Value=3]<br>- Not Often [Value=4]<br>- Never [Value=5]<br>- N/A [Value=6]<br>- I prefer not to answer this question [Value=7]	--Select-- - Very Often [Value=1]<br>- Often [Value=2]<br>- Sometimes [Value=3]<br>- Not Often [Value=4]<br>- Never [Value=5]<br>- N/A [Value=6]<br>- I prefer not to answer this question [Value=7]
***24)** When buying a ____ to use, how often do you look for labels indicating the product is made with natural, non-toxic or eco-friendly ingredients?	--Select--	--Select--	--Select--

	a.) cleaning products	b.) sanitizing products	c.) disinfection products
***25)** Before purchasing the _____, do you look it up on a healthy product app or website?	--Select-- - Yes [Value=1]<br>- No [Value=2]<br>- N/A [Value=3]<br>- I prefer not to answer this question [Value=4]	--Select-- - Yes [Value=1]<br>- No [Value=2]<br>- N/A [Value=3]<br>- I prefer not to answer this question [Value=4]	--Select-- - Yes [Value=1]<br>- No [Value=2]<br>- N/A [Value=3]<br>- I prefer not to answer this question [Value=4]

————————————————Page Break————————————————

Place of Work and Time Worked

***26)** On average, how many days per week do you work at this school?

--Select--

- 1 [Value=1]
- 2 [Value=2]
- 3 [Value=3]
- 4 [Value=4]
- 5 [Value=5]
- 6 [Value=6]
- 7 [Value=7]
- I prefer not to answer [Value=20]
- Other (please specify) [Value=19]

Other: ______________________

***27)** On average, how many hours do you work per day in your assigned classroom/office at this school each week?

--Select--

- 1 [Value=1]
- 2 [Value=2]
- 3 [Value=3]
- 4 [Value=4]
- 5 [Value=5]
- 6 [Value=6]
- 7 [Value=7]
- 8 [Value=8]
- 9 [Value=9]
- 10 [Value=10]
- 11 [Value=11]
- 12 [Value=12]
- I prefer not to answer [Value=20]
- Other (please specify) [Value=19]

Other: ______________________

————————————————Page Break————————————————

Safety & Health (of Employee)

***28)** How important of a concern is your safety and health while working at your school?

- ◯ Very Important [Value=1]
- ◯ Important [Value=2]
- ◯ Neutral [Value=3]
- ◯ Unimportant [Value=4]
- ◯ Very Unimportant [Value=5]
- ◯ I prefer not to answer [Value=6]
- ◯ N/A [Value=7]

***29)** How important of a concern is your safety and health while working in your assigned classroom/office?

- ◯ Very Important [Value=1]
- ◯ Important [Value=2]
- ◯ Neutral [Value=3]
- ◯ Unimportant [Value=4]
- ◯ Very Unimportant [Value=5]
- ◯ I prefer not to answer [Value=6]
- ◯ N/A [Value=7]

————————————Page Break————————————

Symptoms (of Employee)

***30)** Have you ever had any of the following respiratory health problems after working in this school: runny nose, itchy/watery eyes, trouble breathing or headache?

- ◯ Yes [Value=1]
- ◯ No [Value=2]
- ◯ I'm not sure [Value=3]
- ◯ I prefer not to answer [Value=4]

Question Logic
If **[Yes]** is selected, then skip to question **[#31]**
If **[No]** is selected, then skip to question **[#32]**
If **[I'm not sure]** is selected, then skip to question **[#32]**
If **[I prefer not to answer]** is selected, then skip to question **[#32]**

————————————Page Break————————————

***31)** Did you need to seek medical attention in response to the severity of any symptoms? (runny nose, itchy/watery eyes, trouble breathing and/or headache)

- ◯ Yes [Value=1]
- ◯ No [Value=2]
- ◯ I prefer not to answer [Value=3]

***32)** If this is your 1st year at this school, did you experience any of these symptoms at your prior school or job? (runny nose, itchy/watery eyes, trouble breathing and/or headache)

- ◯ Yes [Value=1]
- ◯ No [Value=2]
- ◯ I'm not sure [Value=3]
- ◯ N/A [Value=5]
- ◯ I prefer not to answer [Value=4]

***33)** Do any of these symptoms disappear if you are away from work more than a day? (runny nose, itchy/watery eyes, trouble breathing and/or headache)

- ◯ Yes [Value=1]
- ◯ No [Value=2]
- ◯ I'm not sure [Value=3]
- ◯ N/A [Value=5]
- ◯ I prefer not to answer [Value=4]

————————————Page Break————————————

Ventilation

***34)** Is there a classroom or shop ventilation system?

- ◯ Yes [Value=1]
- ◯ No [Value=2]
- ◯ I'm not sure [Value=5]
- ◯ N/A [Value=3]
- ◯ I prefer not to answer [Value=4]

Can the windows be opened in the school room you spend most time in?

*35)
- ◯ Yes [Value=1]
- ◯ No [Value=2]
- ◯ I'm not sure [Value=5]
- ◯ N/A [Value=3]
- ◯ I prefer not to answer [Value=4]

*36) Is there a ceiling fan in the salon?
- ◯ Yes [Value=1]
- ◯ No [Value=2]
- ◯ I'm not sure [Value=5]
- ◯ N/A [Value=3]
- ◯ I prefer not to answer [Value=4]

————————————Page Break————————————

Personal Protective Equipment (Products Use)

*37) When performing cleaning/disinfection services, do you wear gloves?
- ◯ Yes [Value=1]
- ◯ No [Value=2]
- ◯ N/A [Value=3]
- ◯ I prefer not to answer [Value=4]

*38) When performing cleaning/disinfection services, do you wear a mask?
- ◯ Yes [Value=1]
- ◯ No [Value=2]
- ◯ N/A [Value=3]
- ◯ I prefer not to answer [Value=4]

————————————Page Break————————————

Training

*39) Was training offered on how to handle cleaning/sanitizing/disinfecting products at your workplace?
- ◯ Yes [Value=1]
- ◯ No [Value=2]
- ◯ I'm not sure [Value=3]
- ◯ N/A [Value=5]
- ◯ I prefer not to answer [Value=4]

Question Logic
If **[Yes]** is selected, then skip to question **[#40]**
If **[No]** is selected, then skip to question **[#41]**
If **[I'm not sure]** is selected, then skip to question **[#41]**
If **[N/A]** is selected, then skip to question **[No logic applied]**
If **[I prefer not to answer]** is selected, then skip to question **[#41]**

————————————Page Break————————————

*40) Was the training in your primary language?
- ◯ Yes [Value=1]
- ◯ No [Value=2]
- ◯ I'm not sure [Value=3]
- ◯ N/A [Value=5]
- ◯ I prefer not to answer [Value=4]

*41) Are you aware of U.S. government resources, from USDOL-OSHA?
- ◯ Yes [Value=1]
- ◯ No [Value=2]

◯ I prefer not to answer [Value=3]

***42)** Are you aware of Hazard Communication Standard and/or Safety Data Sheets?
- ◯ Yes [Value=1]
- ◯ No [Value=2]
- ◯ I prefer not to answer [Value=3]

***43)** Are you aware of NJ Safe Schools' Cosmetology/Right to Know website pages?
- ◯ Yes [Value=1]
- ◯ No [Value=2]
- ◯ I prefer not to answer [Value=3]

————————————————Page Break————————————————

Personal Nutrition

***44)** How often do you now eat processed snacks/foods?
- ◯ Rarely [Value=1]
- ◯ Daily [Value=2]
- ◯ Weekly [Value=3]
- ◯ Monthly [Value=4]
- ◯ N/A [Value=5]
- ◯ I prefer not to answer [Value=6]

How often do you eat from (take-out) and/or at restaurants, for:

	Daily	Three or more times a week	One to two times a week	N/A	I prefer not to answer
***45)** Breakfast?	◯ [Value=1]	◯ [Value=2]	◯ [Value=3]	◯ [Value=4]	◯ [Value=5]
***46)** Lunch?	◯ [Value=1]	◯ [Value=2]	◯ [Value=3]	◯ [Value=4]	◯ [Value=5]
***47)** Dinner?	◯ [Value=1]	◯ [Value=2]	◯ [Value=3]	◯ [Value=4]	◯ [Value=5]

————————————————Page Break————————————————

Personal Sleep Hygiene

***48)** How many hours do you sleep per night on average on regular school days?

--Select--

- Less than 5 hours [Value=1]
- 5 - 6 hours [Value=2]
- 7 - 8 hours [Value=3]
- 9 hours or more [Value=4]
- I prefer not to answer [Value=5]

***49)** How many hours do you sleep per night on average on weekends and holidays/vacation days?

--Select--

- Less than 5 hours [Value=1]
- 5 - 6 hours [Value=2]
- 7 - 8 hours [Value=3]
- 9 hours or more [Value=4]
- I prefer not to answer [Value=5]

Please answer yes, no or I prefer to not answer to the following:

	Yes	No	I'm not sure	I prefer not to answer
***50)** I wake up in middle of night.	◯ [Value=1]	◯ [Value=2]	◯ [Value=4]	◯ [Value=3]

*51) I find it difficult to wake up in the morning on a work/school day.	◯ [Value=1]	◯ [Value=2]	◯ [Value=4]	◯ [Value=3]

——————————————Page Break——————————————

Personal Physical Health

*52) I regularly use PPE or personal protective equipment (in the previous school year 2020-2021).

◯ Yes [Value=1] ◯ No [Value=2] ◯ I'm not sure [Value=3] ◯ I prefer not to answer [Value=4]

*53) I regularly use PPE or personal protective equipment (in the current school year 2021-2022).

◯ Yes [Value=1] ◯ No [Value=2] ◯ I'm not sure [Value=4] ◯ I prefer not to answer [Value=3]

Question Logic
If **[Yes]** is selected, then skip to question **[after #53, Matrix Text]** (See "Edit Logic" for details)
If **[No]** is selected, then skip to question **[#57]**
If **[I'm not sure]** is selected, then skip to question **[#57]**
If **[I prefer not to answer]** is selected, then skip to question **[#57]**

——————————————Page Break——————————————

Do you use the following forms of PPE to protect yourself?

	Yes	No	I'm not sure	I prefer not to answer
*54) Mask/face coverings?	◯ [Value=1]	◯ [Value=2]	◯ [Value=3]	◯ [Value=4]
*55) Gloves?	◯ [Value=1]	◯ [Value=2]	◯ [Value=3]	◯ [Value=4]
*56) Protective eyewear?	◯ [Value=1]	◯ [Value=2]	◯ [Value=3]	◯ [Value=4]

*57) Did you maintain consistent aerobic exercise habits during the course of the COVID-19 pandemic?

◯ Yes [Value=1] ◯ No [Value=2] ◯ I'm not sure [Value=3] ◯ I prefer not to answer [Value=4]

*58) Have you maintained similar aerobic exercise habits during the pandemic when compared to before the pandemic?

◯ Yes [Value=1] ◯ No [Value=2] ◯ I'm not sure [Value=3] ◯ I prefer not to answer [Value=4]

Question Logic
If **[Yes]** is selected, then skip to question **[#59]**
If **[No]** is selected, then skip to question **[after #59, Text]** (See "Edit Logic" for details)
If **[I'm not sure]** is selected, then skip to question **[after #59, Text]** (See "Edit Logic" for details)
If **[I prefer not to answer]** is selected, then skip to question **[after #59, Text]** (See "Edit Logic" for details)

——————————————Page Break——————————————

*59) Was there an increase or decrease in your aerobic exercise habits during the pandemic when compared to before the pandemic?

◯ Increase [Value=1] ◯ Decrease [Value=2] ◯ I'm not sure [Value=3] ◯ I prefer not to answer [Value=4]

——————————————Page Break——————————————

Personal Mental Health

In your opinion, who is responsible for supporting the mental well-being of each of the following:

	other school educational professionals, including	school guidance/academic counselors	school health and behavioral health staff, including	community health and behavioral health providers	N/A	I prefer not to answer

	administrators and staff		school psychologists			
*60) School educational professionals	◯ [Value=1]	◯ [Value=2]	◯ [Value=3]	◯ [Value=4]	◯ [Value=5]	◯ [Value=6]
*61) O&M staff and other types of staff	◯ [Value=1]	◯ [Value=2]	◯ [Value=3]	◯ [Value=4]	◯ [Value=5]	◯ [Value=6]
*62) Students	◯ [Value=1]	◯ [Value=2]	◯ [Value=3]	◯ [Value=4]	◯ [Value=5]	◯ [Value=6]

Do you know someone who had a positive test result for SARS-CoV-2 (COVID-19)?

	Yes	No	I'm not sure	I prefer not to answer
*63) Yourself?	◯ [Value=1]	◯ [Value=2]	◯ [Value=3]	◯ [Value=4]
*64) An immediate family member?	◯ [Value=1]	◯ [Value=2]	◯ [Value=3]	◯ [Value=4]
*65) An extended family member?	◯ [Value=1]	◯ [Value=2]	◯ [Value=3]	◯ [Value=4]
*66) A friend or work colleague?	◯ [Value=1]	◯ [Value=2]	◯ [Value=3]	◯ [Value=4]

Do you know someone who has died from COVID-19?

	Yes	No	I'm not sure	I prefer not to answer
*67) An immediate family member?	◯ [Value=1]	◯ [Value=2]	◯ [Value=3]	◯ [Value=4]
*68) An extended family member?	◯ [Value=1]	◯ [Value=2]	◯ [Value=3]	◯ [Value=4]
*69) A friend or work colleague?	◯ [Value=1]	◯ [Value=2]	◯ [Value=3]	◯ [Value=4]

——————————————Page Break——————————————

*70) Would you recommend the available mental health services to a colleague?

- ◯ Yes [Value=1]
- ◯ No [Value=2]
- ◯ I'm not sure [Value=3]
- ◯ N/A [Value=5]
- ◯ I prefer not to answer [Value=4]

*71) If you have used the mental health services in the school before, on a scale of 1-10 with 10 being the most satisfied, how satisfied were you with the services?

--Select--

- 1 [Value=1]
- 2 [Value=2]
- 3 [Value=3]
- 4 [Value=4]
- 5 [Value=5]
- 6 [Value=6]
- 7 [Value=7]
- 8 [Value=8]
- 9 [Value=9]
- 10 [Value=10]
- I prefer not to answer [Value=11]

72) As a teacher, please select the mental health supports you would like to see at your school as schools transition back to full-time in-person learning? Please select/choose all that apply to you at this time.

- ☐ Improve mental health literacy with psychological first aid or training in how to talk about mental health. [Checked=1]
- ☐ Increase availability of mental health and counseling services. [Checked=1]
- ☐ Expanding virtual counseling services and tele-health services. [Checked=1]
- ☐ Involvement in discussions about what might change and what they need. [Checked=1]
- ☐ Consistent communication to reduce uncertainty and build emotional support. [Checked=1]
- ☐ I prefer not to answer [Checked=1]
- ☐ Other (please specify) [Checked=1]

73) Self-care means taking the time to do things that help you live well and improve both your physical health and mental health. What self-care practices have you implemented in your day-to-day life since the beginning of the COVID-19 pandemic?

- ☐ Getting regular exercise. [Checked=1]
- ☐ Eating healthy. [Checked=1]
- ☐ Staying hydrated. [Checked=1]
- ☐ Making sleep a priority. Stick to a schedule, and make sure you're getting enough sleep. [Checked=1]
- ☐ Trying/practicing a relaxing activity. [Checked=1]
- ☐ Practicing gratitude and positivity. [Checked=1]
- ☐ Staying connected with friends or family members. [Checked=1]
- ☐ I prefer not to answer [Checked=1]
- ☐ Other (please specify) [Checked=1]

———————————————Page Break———————————————

Demographics

***74)** County
Please select the NJ county of your school district/school.
Note: If you work for State of NJ agency (e.g., NJDCF, NJDOE), select "statewide" or last option below.

- ◯ Atlantic [Value=3]
- ◯ Bergen [Value=4]
- ◯ Burlington [Value=5]
- ◯ Camden [Value=6]
- ◯ Cape May [Value=7]
- ◯ Cumberland [Value=8]
- ◯ Essex [Value=9]
- ◯ Gloucester [Value=10]
- ◯ Hudson [Value=11]
- ◯ Hunterdon [Value=12]
- ◯ Mercer [Value=13]
- ◯ Middlesex [Value=14]
- ◯ Monmouth [Value=15]
- ◯ Morris [Value=16]
- ◯ Ocean [Value=17]
- ◯ Passaic [Value=18]
- ◯ Salem [Value=19]
- ◯ Somerset [Value=20]
- ◯ Sussex [Value=21]
- ◯ Union [Value=22]
- ◯ Warren [Value=23]
- ◯ Statewide [Value=2]

75) Race and Ethnicity
Please choose the best option from the list below (listed in alphabetical order), or "other" (write in).

- ☐ American Indian or Alaskan Native [Checked=1]
- ☐ Middle Eastern/North African [Checked=1]
- ☐ Native Hawaiian or Other Asian-Pacific Islander [Checked=1]
- ☐ Hispanic Asian [Checked=1]
- ☐ Hispanic Black [Checked=1]
- ☐ Hispanic White [Checked=1]
- ☐ Non-Hispanic Asian [Checked=1]
- ☐ Non-Hispanic Black [Checked=1]
- ☐ Non-Hispanic White [Checked=1]
- ☐ I prefer not to answer [Checked=1]
- ☐ Other (please specify) [Checked=1]

***76)** Gender

- ◯ Male [Value=1]
- ◯ Female [Value=2]
- ◯ Transgender Woman [Value=5]
- ◯ Transgender Man [Value=6]
- ◯ Non-binary [Value=8]
- ◯ I prefer not to answer [Value=3]
- ◯ Other [Value=4]

[ ]

***77)** Birth Year

--Select--

- 2000 [Value=1]
- 1999 [Value=2]
- 1998 [Value=3]
- 1997 [Value=4]
- 1996 [Value=5]
- 1995 [Value=6]
- 1994 [Value=7]
- 1993 [Value=8]
- 1992 [Value=9]
- 1991 [Value=10]
- 1990 [Value=11]
- 1989 [Value=12]
- 1988 [Value=13]
- 1987 [Value=14]
- 1986 [Value=15]
- 1985 [Value=16]
- 1984 [Value=17]
- 1983 [Value=18]
- 1982 [Value=19]
- 1981 [Value=20]
- 1980 [Value=21]
- 1979 [Value=22]
- 1978 [Value=23]
- 1977 [Value=24]
- 1976 [Value=25]
- 1975 [Value=26]
- 1974 [Value=27]
- 1973 [Value=28]
- 1972 [Value=29]
- 1971 [Value=30]
- 1970 [Value=31]
- 1969 [Value=32]
- 1968 [Value=33]
- 1967 [Value=34]
- 1966 [Value=35]
- 1965 [Value=36]
- 1964 [Value=37]
- 1963 [Value=38]
- 1962 [Value=39]
- 1961 [Value=40]
- 1960 [Value=41]
- 1959 [Value=42]
- 1958 [Value=43]
- 1957 [Value=44]
- 1956 [Value=45]
- 1955 [Value=46]
- 1954 [Value=47]
- 1953 [Value=48]
- 1952 [Value=49]
- 1951 [Value=50]
- 1950 [Value=51]
- Other (please specify) [Value=52]

Other:

[ ]

————————————————Page Break————————————————

***78)** Number of Years Teaching in NJ

--Select--

- 1 [Value=1]
- 2 [Value=2]
- 3 [Value=3]
- 4 [Value=4]
- 5 [Value=5]
- 6 [Value=6]

- 7 [Value=7]
- 8 [Value=8]
- 9 [Value=9]
- 10 [Value=10]
- 11 [Value=11]
- 12 [Value=12]
- 13 [Value=13]
- 14 [Value=14]
- 15 [Value=15]
- 16 [Value=16]
- 17 [Value=17]
- 18 [Value=18]
- 19 [Value=19]
- 20 [Value=20]
- 21 [Value=21]
- 22 [Value=22]
- 23 [Value=23]
- 24 [Value=24]
- 25 [Value=25]
- 26 [Value=26]
- 27 [Value=27]
- 28 [Value=28]
- 29 [Value=29]
- 30 [Value=30]
- 31 [Value=31]
- 32 [Value=32]
- 33 [Value=33]
- 34 [Value=34]
- 35 [Value=35]
- Other (please specify) [Value=36]

Other:

***79)** Number of Years of Teaching Overall

--Select--

- 1 [Value=1]
- 2 [Value=2]
- 3 [Value=3]
- 4 [Value=4]
- 5 [Value=5]
- 6 [Value=6]
- 7 [Value=7]
- 8 [Value=8]
- 9 [Value=9]
- 10 [Value=10]
- 11 [Value=11]
- 12 [Value=12]
- 13 [Value=13]
- 14 [Value=14]
- 15 [Value=15]
- 16 [Value=16]
- 17 [Value=17]
- 18 [Value=18]
- 19 [Value=19]
- 20 [Value=20]
- 21 [Value=21]
- 22 [Value=22]
- 23 [Value=23]
- 24 [Value=24]
- 25 [Value=25]
- 26 [Value=26]
- 27 [Value=27]
- 28 [Value=28]
- 29 [Value=29]
- 30 [Value=30]
- 31 [Value=31]
- 32 [Value=32]
- 33 [Value=33]
- 34 [Value=34]
- 35 [Value=35]
- Other (please specify) [Value=36]

Other:

***80)**

What is the highest education degree completed (choose from options presented)?

- ◯ Bachelor's degree (BA, BS or AB) [Value=1]
- ◯ Master's degree (e.g., MA, MS, M.Ed) [Value=2]
- ◯ Doctoral degree (e.g., DrPH, Ed.D, PhD, PsychD, ScD) [Value=3]
- ◯ Other (please specify) [Value=5]

Other:

How many years of post secondary education (after high school) have you completed?

***81)** --Select--

- 1 [Value=1]
- 2 [Value=2]
- 3 [Value=3]
- 4 [Value=4]
- 5 [Value=5]
- 6 [Value=6]
- 7 [Value=7]
- 8 [Value=8]
- 9 [Value=9]
- 10 [Value=10]
- 11 [Value=11]
- 12 [Value=12]
- 13+ [Value=13]
- Other (please specify) [Value=14]

Other:

Skip Logic
Skip to survey **[#194980]**, question **[#1]**

——————————————Skip Logic——————————————

——————————————Automatic Page Break——————————————

Late 2022/2022-2023 School Year Follow-up (v.1 Aug2021) "Rutgers NJ Safe Schools Program New Work-Based Learning Supervising Teachers Cohort - Survey

Thank you!

For maximum confidentiality, please close this window.

Copyright © 2001-2023 PsychData®, LLC. All rights reserved.
